# The price of altruism, and the limits of scientific inquiry

**Published:** 2011-01-10

**Authors:** Oren Harman

**Affiliations:** 1 Bar-Ilan University, Ramat Gan, 52900, Israel

## 

The origin of kindness is a mystery. Where do giving and altruism come from: were they inherited on the wings of natural selection – a gift bestowed upon us via the inching, evolutionary march of sacrificial amoeba, selfless penguins, and charitable baboons? Or is altruism a unique refinement, a singular human triumph over ‘nature bloody in tooth in claw’? Darwin called this his greatest single riddle and ever since thinkers have tried to crack it.

One hundred and fifty years of attempts to crack the mystery of altruism can teach us a lot about how prior ideological commitments find their way directly into the scientific enterprise. Beginning with the debate between “Darwin’s bulldog”, Thomas Henry Huxley, and the Russian anarchist prince Peter Kropotkin, the history of such attempts tells a story of direct contact, and mutual interplay, between social, political and scientific thought. Whether it is the Chicago ecologists of the 1920s through 1940s, arguing for group selection and the “superorganism” as Fascism tore through Europe, or John von Neumann and the progenitors of Game Theory touting a belief in the utter selfishness of the “maximizing agent” as they modeled games on their swivel chairs at RAND, theories of altruism and cooperation have been closely linked to scientists’ own understanding of the moral good and its social consequences.

Neurogenetics and fMRI studies are showing today that kindness may be located in our genes and in particular parts of our brain. Coupled to animal behavior studies, brain damage studies, evolutionary psychology logic, and mathematical modeling, they are supposedly pointed in the direction of cracking the altruism mystery. But precisely for this reason, we need to remember the fate of George Price, who will be the focus of this presentation. The achievements and tragedy of this hitherto forgotten American genius chemist-turned-evolutionist-turned-homeless vagabond, I will argue, provide salutary lessons regarding our understanding of altruism, as well as the limits of scientific inquiry. “Even if all possible scientific questions be answered”, the twentieth century philosopher Ludwig Wittgenstein wrote, “The problems of life have still not been touched at all. Of course there is then no question left, and just this is the answer”. Price's incredible life story will be presented to argue this point.

